# Functional and genetic analysis of choroid plexus development in zebrafish

**DOI:** 10.3389/fnins.2014.00364

**Published:** 2014-11-10

**Authors:** Hannah E. Henson, Chaithanyarani Parupalli, Bensheng Ju, Michael R. Taylor

**Affiliations:** ^1^Chemical Biology and Therapeutics, St. Jude Children's Research HospitalMemphis, TN, USA; ^2^Integrated Program in Biomedical Sciences, College of Graduate Health Sciences, University of Tennessee Health Science CenterMemphis, TN, USA; ^3^Pharmaceutical Sciences Division, School of Pharmacy, University of Wisconsin-MadisonMadison, WI, USA

**Keywords:** choroid plexus, zebrafish, forward genetic screen, Claudin 5, transporters, fluorescein, genetic mapping

## Abstract

The choroid plexus, an epithelial-based structure localized in the brain ventricle, is the major component of the blood-cerebrospinal fluid barrier. The choroid plexus produces the cerebrospinal fluid and regulates the components of the cerebrospinal fluid. Abnormal choroid plexus function is associated with neurodegenerative diseases, tumor formation in the choroid plexus epithelium, and hydrocephaly. In this study, we used zebrafish (*Danio rerio*) as a model system to understand the genetic components of choroid plexus development. We generated an enhancer trap line, *Et(cp:EGFP)*^*sj*2^, that expresses enhanced green fluorescent protein (EGFP) in the choroid plexus epithelium. Using immunohistochemistry and fluorescent tracers, we demonstrated that the zebrafish choroid plexus possesses brain barrier properties such as tight junctions and transporter activity. Thus, we have established zebrafish as a functionally relevant model to study choroid plexus development. Using an unbiased approach, we performed a forward genetic dissection of the choroid plexus to identify genes essential for its formation and function. Using *Et(cp:EGFP)*^*sj*2^, we isolated 10 recessive mutant lines with choroid plexus abnormalities, which were grouped into five classes based on GFP intensity, epithelial localization, and overall choroid plexus morphology. We also mapped the mutation for two mutant lines to chromosomes 4 and 21, respectively. The mutants generated in this study can be used to elucidate specific genes and signaling pathways essential for choroid plexus development, function, and/or maintenance and will provide important insights into how these genetic mutations contribute to disease.

## Introduction

The choroid plexus consists of polarized epithelial cells projecting into the brain ventricle to create a blood-cerebrospinal fluid barrier between fenestrated capillaries and the cerebrospinal fluid (Wolburg and Paulus, 2010). These epithelial cells are modified ependymal cells derived from embryonic neuroepithelial cells that form intercellular tight junctions and express multiple families of transporters that create both a physical and chemical barrier. The choroid plexus also plays an important role in the production and secretion of the cerebrospinal fluid and the regulation of central nervous system homeostasis. Although the choroid plexus of the lateral brain ventricles was documented by Herophilus, an ancient Greek physician (Dohrmann, 1970; Dziegielewska et al., 2001), there has been little research aimed toward identifying the genetic pathways essential for choroid plexus formation and function. Previous studies have classified choroid plexus development by observing modifications in cell morphology, measuring glycogen content as it matures, and examining gene expression (Dohrmann, 1970; Strazielle and Ghersi-Egea, 2000; Dziegielewska et al., 2001; Kratzer et al., 2012, 2013; Liddelow et al., 2014). Signaling pathways such as Sonic hedgehog (Huang et al., 2009; Nielsen and Dymecki, 2010), BMP (Currle et al., 2005), and Notch (Irvin et al., 2004) have been found to contribute to choroid plexus formation. However, how these pathways interact with one another to generate the choroid plexus and the downstream components involved in its development, function, and maintenance are unknown. Understanding normal development and function in the choroid plexus is crucial to unraveling how these mechanisms become altered in diseases. The choroid plexus has been associated with various disease conditions, including choroid plexus tumors (Wolburg and Paulus, 2010), hydrocephalus (Wodarczyk et al., 2009), and neurodegenerative diseases such as Alzheimer's disease (Alvira-Botero and Carro, 2010) and multiple sclerosis (Vercellino et al., 2008). Identifying altered genes and signaling pathways in the choroid plexus that cause disease initiation or progression can help determine potential therapeutic targets for treatment.

Models used to study the choroid plexus have included (1) mice, because of their similarity to the human choroid plexus in gene expression patterns (Janssen et al., 2013); (2) marsupials, because post-natal lateral ventricle development occurs outside of the womb (Liddelow et al., 2010); and (3) *in vitro* cell culture systems to study choroid plexus transport (Monnot and Zheng, 2013). However, these models do not provide real-time, *in vivo* developmental information. We propose, as have others (Bill et al., 2008; Garcia-Lecea et al., 2008), that zebrafish are a suitable vertebrate model to analyze and genetically dissect choroid plexus development and function *in vivo*, as they offer several advantages such as having central nervous system structures similar to those in mammals, rapid *ex utero* development, transparency, large numbers of offspring, and genetic tractability (Goldsmith and Jobin, 2012).

Previous studies have examined the role of Shh and Notch signaling in choroid plexus development using zebrafish (Bill et al., 2008; Garcia-Lecea et al., 2008). However, these studies did not examine functional choroid plexus barrier properties such as tight junctions and transporters. Here, we generated an enhancer trap line, *Et*(*cp:EGFP*)^*sj*2^, that expresses enhanced green fluorescent protein (EGFP) in epithelial cells of the diencephalic choroid plexus and myelencephalic choroid plexus. We used this enhancer trap line to visualize choroid plexus development *in vivo* and demonstrated that several components of the zebrafish choroid plexus are comparable to human. We also performed a small-scale ENU-mutagenesis screen and identified 10 recessive mutant lines. These mutants were classified into five categories on the basis of EGFP expression and choroid plexus morphology. We also genetically mapped the mutations from two mutant lines using bulked-segregant analysis. The cloning and characterization of these mutants will provide important insights into the genetic pathways that regulate formation and function of the choroid plexus in health and disease.

## Materials and methods

### Fish lines and maintenance

Zebrafish were maintained at 28.5° on a 14 h light/dark cycle. Embryos used for imaging or for screening F_3_ larvae were collected in egg water (0.03% Instant Ocean) containing 0.02% methylene blue and treated at 24 h post-fertilization (hpf) with 0.003% 1-phenyl-2-thiourea (PTU) (Sigma) to prevent pigment formation. Zebrafish were maintained in accordance with established protocols and all experiments were approved by the St. Jude Children's Research Hospital Institutional Animal Care and Use Committee.

### Generation of the *Et(cp:EGFP)*^*sj*2^ line

To generate the *Et(cp:EGFP)*^*sj*2^ line, EGFP was released from the pEGFP-N1 vector (Clontech) by *Bam*HI and *Not*I digestion and inserted into the pTRE-Tight vector (Clontech). The *TRE-tight:EGFP* fragment was released by *Xho*I and *Cla*I digestion and inserted into the pT2AL200R150G vector (Urasaki et al., 2006). Approximately 30 pg of the resulting T2 (*TRE-tight:EGFP)* plasmid DNA was co-injected with 30 pg of *in vitro* transcribed Tol2 transposase mRNA into single-cell embryos of the AB strain. The embryos were raised to adults and screened for germline transmission by examining their offspring for EGFP expression.

### Immunohistochemistry (IHC)

Larvae were anesthetized in 0.02% tricaine and fixed in 4% paraformaldehyde (PFA) (Electron Microscopy Sciences) at 4° overnight and washed the next day in 1× phosphate buffered serum (PBS) (Calbiochem). Samples were sunk in 30% sucrose/PBS at 4° overnight. Larval 1-month-old brain tissues, and adult brains were embedded in Optimal Cutting Temperature (O.C.T.) Compound (Tissue-Tek), frozen on dry ice, and stored at −80°. Tissues were sectioned using a Leica CM 1950 cryostat. Sections were washed in PBS for 5 min followed by three 5 min washes in PBST [PBS/0.03% Triton X-100 (Sigma)]. Sections were incubated in blocking buffer [PBST with 5% goat serum (Gibco) and 1% BSA (Sigma)] for 3 h at room temperature. Primary antibodies were incubated at 4° overnight followed by secondary antibody incubation for 2 h at room temperature. Sections were washed in PBST four times for 15 min after primary and secondary antibody incubations. Primary antibodies included rabbit anti-GFP (1:500; Invitrogen) and mouse anti-Zpr1 (1:100; ZIRC). Secondary antibodies included Alexa Fluor goat anti-rabbit 488 (1:200; Invitrogen) and Alexa Fluor goat anti-mouse 555 (1:200; Invitrogen). Antibody dilutions were prepared in blocking buffer. Sections were counterstained with 1 μg/mL DAPI (Roche) for 1 min, rinsed briefly in PBS, and mounted with Fluoromount (Electron Microscopy Sciences). Images were taken on a Nikon AZ100 microscope and analyzed using NIS-Elements AR 3.2 software.

### Whole-mount IHC

Embryos were incubated in egg water with 0.003% PTU to prevent pigment formation. At 4 and 6 days post-fertilization (dpf), larvae were anesthetized in 0.02% tricaine and fixed in 4% PFA overnight. The next day, samples were washed in 1× PBS followed by 1× PBST and treated with 20 μg/mL Proteinase K (New England Biolabs) for 15 min. The reaction was stopped by adding 10% lamb serum (Gibco) followed by additional washes in PBST. Samples were blocked with 10% lamb serum for 1–4 h and incubated in primary antibody at 4° overnight. Samples were washed the next day in PBST and incubated in secondary antibody at 4° overnight. Antibodies used included rabbit anti-GFP (1:100; Invitrogen), mouse anti-Zpr1 (1:50; ZIRC), mouse anti-Cldn5 (1:100; Invitrogen), Alexa Fluor goat anti-rabbit 488 (1:200; Invitrogen), and Alexa Fluor goat anti-mouse 555 (1:200; Invitrogen). Additional washes in PBST were done the following day and stored in 1× PBS. Samples were embedded in 0.8% low-melting point agarose (Invitrogen) made in 1× PBS and imaged on Nikon C1Si laser scanning confocal microscope. Z-stacks were compiled to create maximum intensity projection images using Nikon NIS-Elements 3.1 imaging software.

### Fluorescent tracer injections

To observe transporter activity in the choroid plexus, *Et*(*cp:EGFP*)^*sj*2^ larvae at 4 dpf were anesthetized in 0.02% tricaine and injected intravenously into the common cardinal vein with approximately 1–2 nL of 100 μM rhodamine 123 (Sigma-Aldrich) using a micromanipulator and a pneumatic picopump (World Precision Instruments). The total blood volume of zebrafish embryos at 2 dpf has been estimated to be 60 nL (Craig et al., 2012), so the blood volume is not significantly altered upon injection. Immediately after the injections, larvae were embedded in 1.2% low-melting-point agarose (Invitrogen) made in egg water. Larvae were imaged on a Nikon C1Si confocal microscope and analyzed using Nikon EZC1 3.91 software.

To study choroid plexus function, Casper larvae (lacking melanocytes and iridophores) (White et al., 2008) were anesthetized in 0.02% tricaine and intravenously injected into the common cardinal vein with approximately 0.3–2 nL of 10 mg/mL rhodamine- and fluorescein-labeled dextrans at 2, 3, and 4 dpf. Fluorescent tracers included a 3-kDa fluorescein dextran, a 10-kDa rhodamine dextran, and a 40-kDa anionic fluorescein dextran (Invitrogen). Immediately after the injections, larvae were laterally embedded in 1.2% low-melting-point agarose made in egg water. To observe tracer permeability into the brain ventricle, time-lapse images were collected every 2 min for 1 h with a Nikon AZ100 microscope equipped with a shutter (Sutter Instruments) and analyzed with Nikon NIS-Elements 3.2 software. *Et*(*cp:EGFP*)^*sj*2^ larvae were also injected intravenously with the 10-kDa rhodamine dextran at 2 and 4 dpf and imaged on a Nikon C1Si confocal microscope with Nikon EZC1 3.91 software.

To visualize brain ventricle morphology in wild-type and mutant *Et*(*cp:EGFP*)^*sj*2^, PTU-treated larvae at 4 dpf were anesthetized and intraventricularly injected with approximately 2 nL of 40-kDa fluorescein-labeled dextran as described previously (Gutzman and Sive, 2009). Larvae were imaged immediately after the injection using a Nikon SMZ1500 epifluorescence stereomicroscope and analyzed with Nikon NIS-Elements 3.1 software. Ventricular injections of rhodamine 123 and fluorescein were performed as described previously (Gutzman and Sive, 2009) in Casper larvae at 4 dpf and imaged on a Nikon C1Si confocal microscope with Nikon EZC1 3.91 software.

### Fluorescein treatment

Casper and *Et*(*cp:EGFP*)^*sj*2^ larvae were incubated for 4 h in the dark with 50 μM fluorescein (Fluka). Larvae were briefly rinsed 12 times in egg water, anesthetized in 0.02% tricaine, and embedded in 1.2% low-melting-point agarose. After 30 min, larvae were imaged using a Nikon SMZ1500 epifluorescence stereomicroscope and analyzed with Nikon NIS-Elements 3.1 software.

### Statistical analysis of tracer permeability

The ratio of the fluorescence intensity in the ventricle to that in the heart was calculated at the 30-min timepoint when the leakiest tracer reached saturation. The ratio was then normalized against the tracer having the greatest mean intensity in the ventricle at 30 min. A One-Way ANOVA test was performed in Microsoft Excel to determine whether there was a significant difference in fluorescent intensity between the tracers and developmental timepoints. A Tukey's *post-hoc* test was performed to determine which tracers were statistically significant to one another at each developmental timepoint. A significance value of α = 0.05 was used, and *P*-values were calculated using the GraphPad software *P*-value calculator. Error bars were based on the mean of 7 observations using standard error.

### N-ethyl-N-nitrosourea (ENU) mutagenesis and forward genetic screening

Twenty *Et(cp:EGFP)*^*sj*2^ males were treated three times with 3 mM ENU (Sigma) for 1 h each at weekly intervals as described previously (Driever et al., 1996). After 1 month, ENU-treated males were mated to *Et*(*cp:EGFP*)^*sj*2^ females to produce the F_1_ generation. F_1_ pairwise crosses were performed to produce F_2_ families. F_2_ families that did not contain at least 4 male/female pairs were sacrificed. Pairwise crosses for each F_2_ family were done at least six times if possible to identify F3 offspring with homozygous recessive mutations. F_2_ pairs deemed heterozygous for a recessive mutation were maintained in miniboxes for use in genetic mapping experiments. To screen for choroid plexus mutants, F_3_ larvae at 4 dpf were anesthetized and visualized using a Nikon SMZ1500 epifluorescence stereomicroscope to identify abnormal GFP expression or patterning in the choroid plexus. A mutant line was confirmed if approximately 25% of the total offspring displayed a choroid plexus phenotype.

### Genomic DNA isolation

Adult zebrafish were anesthetized in 0.02% tricaine (Sigma-Aldrich) and tail clipped. As the fish recovered in system water, the tail clip was immediately immersed into 50 μL digest buffer (0.2 M NaCl, 10 mM Tris 8.0, 5 mM EDTA, 0.1% SDS, 0.1 mg/ml Proteinase K). Samples were incubated overnight at 50°. After brief centrifugation, 450 μL of 100% ethanol (EtOH) added. Samples were centrifuged at 21,130 *g* for 10 min, the supernatant was removed, and 500 μL of 70% EtOH added to wash the pellet. Samples were centrifuged at 21,130 *g* or 15,000 rpm for 5 min. After removing the supernatant, the samples were stored at room temperature for about 30 min to evaporate excess EtOH. The DNA pellets were resuspended with 50 μL of TE buffer (10 mM Tris and 1 mM EDTA, pH 8.0).

For zebrafish larvae, wild-type and mutants were anesthetized, separated into 1.5 mL eppendorf tubes, and immersed in 1 mL 100% methanol (MeOH) (Sigma-Aldrich). Samples were stored at −20° overnight. The following day, individual wild-type and mutant larvae were transferred into 0.5 mL tubes and the excess MeOH removed. Once all the MeOH evaporated, 10 μL of digest buffer (same as above) was added to each tube. Samples were incubated overnight at 50°. DNA was isolated as described above for adults except the volume of 100% EtOH and 70% EtOH was 180 μL and the volume of TE buffer was 25 μL.

### Genetic mapping

After identifying a mutant line, the *Et(cp:EGFP)*^*sj*2^ F_2_ heterozygous parents were each outcrossed to the polymorphic TL strain. From this outcross, we identified AB/TL hybrids heterozygous for the recessive mutation by screening their offspring. The wild-type and mutant offspring from these heterozygous hybrids were collected and their DNA was extracted (see above). A genome scan using 192 polymorphic Z-markers was performed with DNA pooled from 30 wild-type and 30 mutant larvae as previously described (Muto et al., 2005). Wild-type and mutant DNA was amplified using the Accuprime Taq DNA Polymerase System (Invitrogen). The resulting PCR products were analyzed for genetic linkage by agarose gel electrophoresis. For fine genetic mapping, individual wild-type and mutant DNA was amplified using Platinum PCR Supermix (Invitrogen). Z-markers used for genetic mapping were acquired from the zebrafish genome database at www.ensembl.org. Additional markers were designed by examining the Ensembl genome browser for polymorphic regions in the genome.

### Live confocal imaging and time-lapse movies

Confocal imaging was performed on a Nikon C1Si confocal microscope and analyzed using Nikon EZC1 3.91 software. Scans of DIC and GFP images were acquired using a 20× objective at 4 μm intervals to create a z-stack maximum intensity projection image. To observe choroid plexus development, larvae were collected at ~30 hpf and prepared for imaging as described above. Z-stack images were acquired every 30 min over a 65 h period. Using NIS-Elements AR 4.0 software, images were smoothed and z-stacks were compiled to create an enhanced depth of focus (EDF) image. To compare choroid plexus development, wild-type and mutant larvae were imaged beginning at 54 hpf with images acquired every 30 min over a 48 h period. Supplemental movies run for the 48 h period except for Supplemental movie 6 which runs for 42 h.

## Results

### Generation and characterization of the zebrafish choroid plexus enhancer trap line *Et(cp:EGFP)*^*sj*2^

In the process of producing a tetracycline-inducible transgenic line, we fortuitously generated the zebrafish choroid plexus line by an enhancer trap effect where EGFP became “trapped” within the genome and is now regulated by an enhancer. To generate this line, we co-injected a TRE-EGFP construct with *Tol2* transposase mRNA into single-cell zebrafish embryos. The TRE-EGFP construct was integrated into an enhancer region that regulates gene expression in the choroid plexus. Using Southern blot analysis, we identified a single TRE-EGFP insertion on chromosome 13 (data not shown). By sequencing genes within this region, we identified the TRE-GFP transgene between two genes *TATA box binding protein* (*tbp*) and *asparaginase-like1* (*asrgl1*), both of which according to zfin.org are not spatially restricted in their mRNA expression patterns. After performing *in situ* hybridization on *asrgl1*, we did not observe expression within the choroid plexus (data not shown). Because we were unable to identify choroid plexus specific genes along this region of Chromosome 13, we concluded that the GFP expression in the choroid plexus is the result of an enhancer trap affect where an enhancer localized upstream or downstream of GFP is regulating GFP expression. The insertion was transmitted to subsequent generations, thereby creating a stable line termed *Et(cp:EGFP)*^*sj*2^.

**Figure 1:**
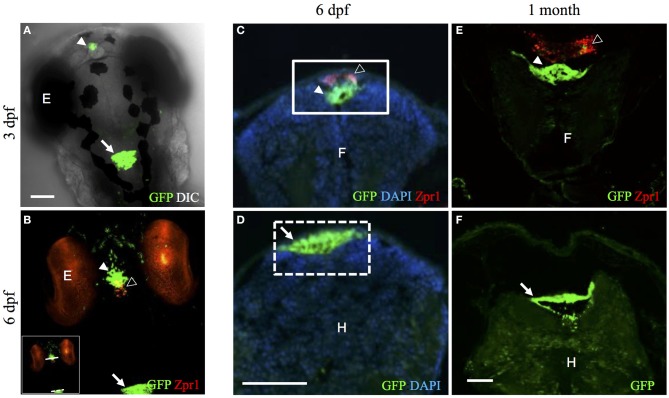
**The *Et(cp:EGFP)*^*sj*2^ line expresses GFP in the diencephalic choroid plexus and myelencephalic choroid plexus. (A)** Confocal image showing the dorsal view of the head of 3 dpf *Et*(*cp:EGFP*)^*sj*2^ larvae expressing GFP in the diencephalic choroid plexus and myelencephalic choroid plexus. **(B)** Whole-mount immunohistochemical staining of *Et(cp:EGFP)*^*sj*2^ larvae at 6 dpf. GFP in green labels the diencephalic choroid plexus and myelencephalic choroid plexus, and Zpr-1 shown in red labels the pineal gland and photoreceptors. The yellow overlay demonstrates the interaction between the pineal gland and diencephalic choroid plexus. The solid and dashed lines across the diencephalic choroid plexus and myelencephalic choroid plexus in the inset image indicate approximate regions where sections were taken for **(C,D)**. **(C)** Transverse section through the diencephalic choroid plexus of 6 dpf *Et*(*cp:EGFP*)^*sj*2^ larvae stained with GFP, DAPI, and Zpr-1. **(D)** Transverse section through the myelencephalic choroid plexus of 6 dpf *Et*(*cp:EGFP*)^*sj*2^ larvae stained with GFP and DAPI. **(E)** Transverse section through the diencephalic choroid plexus of a 1-month-old *Et*(*cp:EGFP*)^*sj*2^ zebrafish stained with GFP and Zpr-1 to label the diencephalic choroid plexus and pineal gland, respectively. **(F)** Transverse section through the myelencephalic choroid plexus of a 1-month-old *Et*(*cp:EGFP*)^*sj*2^ zebrafish stained with GFP. Abbreviations: diencephalic CP, diencephalic choroid plexus; mCP, myelencephalic CP; GFP, green fluorescent protein; DAPI, 4′,6-diamidino-2-phenylindole; E, eye; F, forebrain; H, hindbrain. For all images: filled arrowhead, diencephalic choroid plexus; open arrowhead, pineal gland; arrow, myelencephalic choroid plexus. Scale bars are 50 μm.

GFP expression was specifically localized to the diencephalic choroid plexus in the third ventricle and the myelencephalic choroid plexus in the fourth ventricle at 3 dpf corresponding to the mammalian choroid plexus in third and fourth ventricles, respectively (Figure 1A). We identified that this integration resulted in EGFP expression in the diencephalic choroid plexus and myelencephalic choroid plexus based on two previously published papers also identifying GFP expression in the diencephalic choroid plexus and myelencephalic choroid plexus of zebrafish (Bill et al., 2008; Garcia-Lecea et al., 2008). Unlike mammals, the zebrafish choroid plexus is dorsal to the brain allowing for easy visualization of choroid plexus development. As mentioned in the two previous zebrafish choroid plexus studies, the myelencephalic choroid plexus in teleosts exists as a layer of GFP positive cells on the dorsal surface of the fourth ventricle and ventral to the outer skin epithelium, while the diencephalic choroid plexus is at the most dorsal portion of the third ventricle (Bill et al., 2008; Garcia-Lecea et al., 2008). This allows choroid plexus development to be easily visualized in the zebrafish because it is not buried inside the brain as in other vertebrates. Similarly as described in the dogfish shark, the myelencephalic choroid plexus lies over the brain ventricle rather than obstructed between the medulla and cerebellum (Villalobos et al., 2002). Other studies on zebrafish brain development describe how the development of the telencephalon folds outward with the proliferating cells on the outside rather than inward as seen in other vertebrates (Schmidt et al., 2013), suggesting the zebrafish has more of an “inside out” morphology. This type of development in zebrafish, termed the eversion model, begins with brain ventricular formation followed by the movement of surrounding neural tissue migrating into the ventricle space and results in an overlying ventricle surrounding the brain tissue (Folgueira et al., 2012). Because brain ventricular formation occurs prior to choroid plexus development, the eversion process may be responsible for this “inside out” morphology.

To gain further insight into the location of the choroid plexus in relation to other central nervous system structures, we used whole-mount immunohistochemistry (IHC) to stain *Et(cp:EGFP)*^*sj*2^ larvae with a Zpr-1 antibody labeling cone photoreceptors of the eye and the pineal gland. In other teleosts and mammals, the pineal gland directly interacts with the choroid plexus of the third ventricle (Omura et al., 1985; Skinner and Malpaux, 1999). Although the diencephalic choroid plexus is distinct from the pineal gland, we found a close association between the two structures, where the diencephalic choroid plexus was found anterior to the pineal gland (Figure 1B). Transverse sections showed the diencephalic choroid plexus positioned beneath the pineal gland (Figure 1C). Additional transverse sections through the myelencephalic choroid plexus showed a cluster of GFP-positive epithelial cells directly beneath the skin surface (Figure 1D). GFP expression in the diencephalic choroid plexus and myelencephalic choroid plexus was retained throughout development and at later stages, as shown in a 1-month-old *Et(cp:EGFP)*^*sj*2^ (Figures 1E,F).

**Figure 2:**
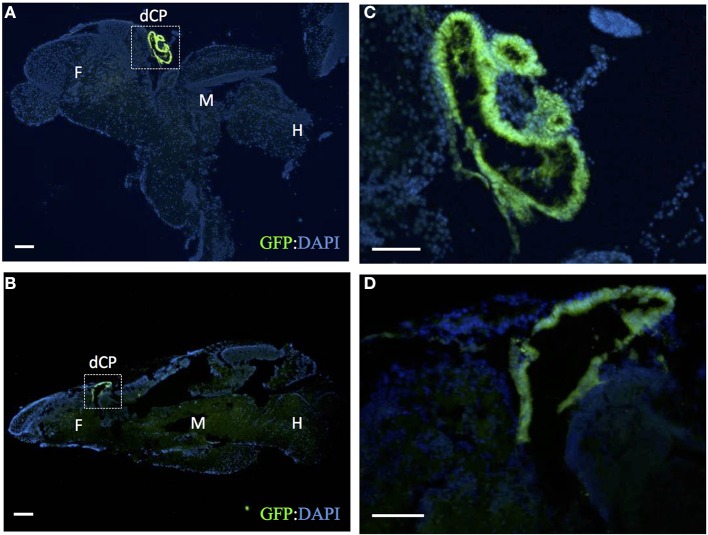
**The adult *Et(cp:EGFP)*^*sj*2^ expresses GFP in the saccus dorsalis**. Sagital sections through adult brains were labeled with rabbit anti-GFP antibody (green) and counterstained with DAPI (blue). **(A,B)** shows GFP expression in the adult CP of the third ventricle taken through two different sagittal planes of the adult brain. Dashed-line boxes refer to magnified images shown in **(C,D)**. dCP: diencephalic CP; F: forebrain; M: midbrain; H: hindbrain. Scale bars are 100 μm.

To further demonstrate that this is indeed the choroid plexus, we cut sections from adult brains and showed expression of GFP in a structure historically referred to as the saccus dorsalis (Figure 2). This structure is homologous to the mammalian choroid plexus of the third ventricle. The saccus dorsalis in rainbow trout (*Salmo gairdneri*), has been described as a folded monolayer of epithelium that develops out of the diencephalic roof plate (Jansen et al., 1976). Because the saccus dorsalis has also been described to partially cover the pineal stalk (Tsuneki, 1986), similar to what we show in Figure 1B, we conclude that this is the choroid plexus of the third ventricle in zebrafish. We also detected GFP expression in the swimbladder (data not shown). The swimbladder consists of epithelial cells and has been compared to the mammalian lung (Winata et al., 2009). Using time-lapse confocal microscopy, GFP expression was detected in a subset of diencephalic choroid plexus cells as early as 30 hpf (see Supplemental Movie 1). We found that the myelencephalic choroid plexus epithelial cells migrated from the outer rhombomeres into the midbrain-hindbrain boundary as described previously (Garcia-Lecea et al., 2008) and also anteriorly from the developing spinal cord. These cells increase in GFP intensity as they coalesce to form a compact structure by approximately 72 hpf. There were no additional changes in choroid plexus morphology by 96 hpf.

### Zebrafish choroid plexus expresses the tight junction protein claudin 5 and possesses transporter activity

**Figure 3:**
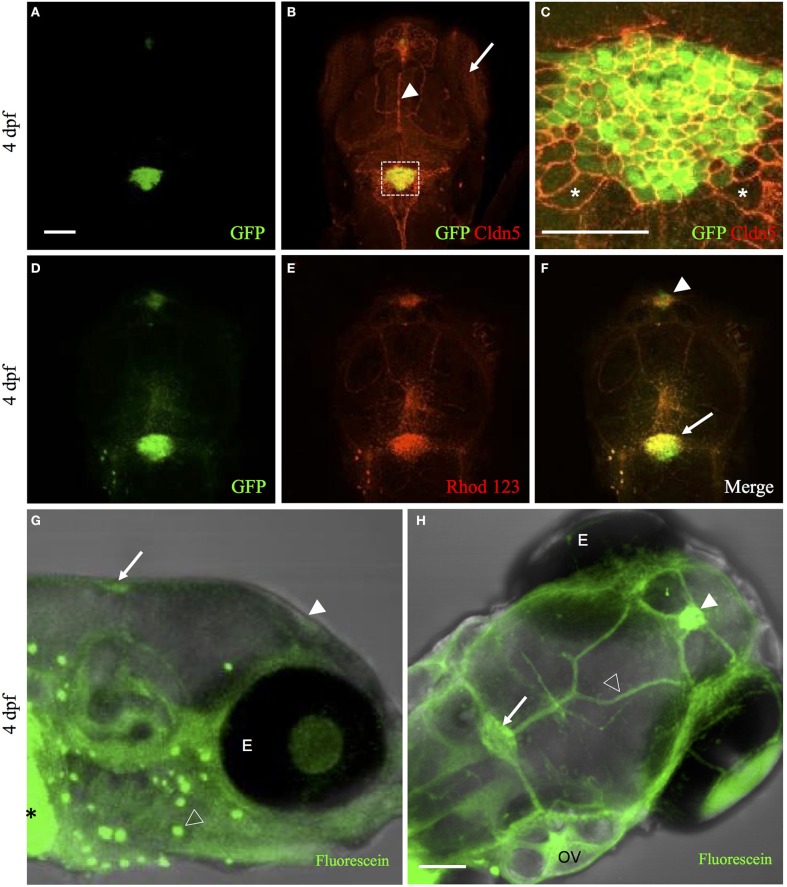
**The zebrafish CP possesses tight junction and transporter properties**. **(A)** Whole-mount immunohistochemical staining of *Et(cp:EGFP)*^*sj*2^ larvae at 4 dpf with GFP labeling the diencephalic choroid plexus and myelencephalic choroid plexus. **(B)** Whole-mount immunohistochemical staining with Claudin 5 antibody (red) showed expression in the eye (arrow), surface vessels (arrowhead), and CP. **(C)** Overlay of GFP and Claudin 5 staining revealed Claudin 5 expression surrounding the perimeter of CPe and ependymal cells. Examples of ependymal cells are shown with an asterisk (^*^). Images in **(A,B)** taken at 20× magnification and (C) at 40× magnification. **(D)** Live imaging of *Et(cp:EGFP)*^*sj*2^ larvae at 4 dpf showed GFP expression in the CP. **(E,F)** Accumulation of rhodamine 123 in the diencephalic choroid plexus (arrowhead) and myelencephalic choroid plexus (arrow). **(G)** Side view of live Casper zebrafish treated at 4 dpf with 50 μM fluorescein for 4 h showed accumulation of fluorescein (represented in green) in the diencephalic choroid plexus (filled arrowhead) and myelencephalic choroid plexus (arrow) along with the gut (asterisk), and lateral line (open arrowhead). **(H)** Similar image as in **(G)** except for dorsal view showing accumulation in the diencephalic choroid plexus (filled arrowhead), myelencephalic choroid plexus (arrow), and overlying vasculature (open arrowhead). Abbreviations: dCP, diencephalic CP; mCP, myelencephalic CP; E, eye; OV; Otic vesicle. Scale bar in A and H is 50 μm. Scale bar in C is 20 μm.

The mammalian choroid plexus expresses many tight-junction proteins such as claudins (Lippoldt et al., 2000; Kratzer et al., 2012), occludin (Lagaraine et al., 2011), and junction adhesion molecules (Lagaraine et al., 2011). These tight junctions create a physical barrier between the blood and cerebrospinal fluid. To demonstrate that the zebrafish choroid plexus express tight junction protein similar to mammals, we studied Claudin 5 (Cldn5) expression because of the availability of an antibody that cross-reacts with zebrafish Cldn5 (Xie et al., 2010; Zhang et al., 2010). As predicted, whole-mount IHC analysis revealed Cldn5 expression at the cell membrane of zebrafish choroid plexus epithelium (Figures 3A–C). Cldn5 expression was also observed in the eyes, blood vessels, and neighboring ependymal cells (Figures 3B,C).

**Figure 4:**
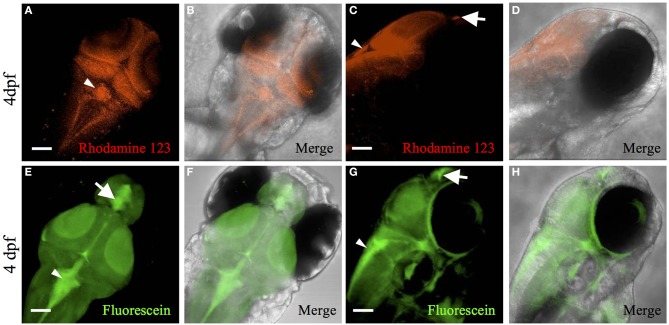
**Rhodamine 123 and fluorescein ventricle injection demonstrates transporter activity in the CP**. Dorsal fluorescent **(A)** and merged (bright-field and Rhodamine 123) **(B)** confocal image of 4 dpf Casper larvae with Rhodamine 123 (red) injection into the brain ventricle. The rhodamine 123 concentrates within the myelencephalic choroid plexus (arrowhead). Rhodamine 123 also fluoresces green and co-localizes with red emission (not shown). Lateral fluorescent **(C)** and merged (bright-field and Rhodamine 123) **(D)** confocal image of 4 dpf Casper larvae with Rhodamine 123 (red) injection into the brain ventricle. The rhodamine 123 concentrates within the myelencephalic choroid plexus (arrowhead) and diencephalic choroid plexus (arrow). Dorsal fluorescent **(E)** and merged (bright-field and fluorescein) **(F)** confocal image of 4 dpf Casper larvae with fluorescein (green) injected into the brain ventricle. Fluorescein concentrates within the myelencephalic choroid plexus (arrowhead) and diencephalic choroid plexus (arrow). Lateral fluorescent **(G)** and merged(bright-field and fluorescein) **(H)** confocal image of 4 dpf Casper larvae with fluorescein (green) injected into the brain ventricle. Fluorescein concentrates within the myelencephalic choroid plexus (arrowhead) and diencephalic choroid plexus (arrow). Abbreviations: mCP, myelencephalic choroid plexus; dCP, diencephalic choroid plexus. Scale bar is 50 μm.

In mice, multidrug resistance transporters, such as MRP1, are expressed on the basolateral side of choroid plexus epithelium and regulate drug accumulation in the cerebrospinal fluid (Wijnholds et al., 2000). In addition to drug transport, MRP1 and MDR1, efflux fluorescent dyes such as rhodamine 123 (Saengkhae et al., 2003). To the best of our knowledge, no previous studies have demonstrated transporter function in zebrafish choroid plexus. To determine whether transporters contribute to the blood-cerebrospinal fluid barrier at the zebrafish choroid plexus, we intravenously injected rhodamine 123 into the *Et(cp:EGFP)*^*sj*2^ line at 4 dpf and observed its accumulation within the choroid plexus epithelium (Figures 3D–F). After injecting rhodamine 123 into the brain ventricle/cerebrospinal fluid, we also observed accumulation in the choroid plexus (Figures 4A–D), indicating that independent of what interface the rhodamine 123 resides (whether blood or cerebrospinal fluid), the dye is transported by the choroid plexus.

Another transporter that is highly expressed in the choroid plexus is organic anion transporter 3 (OAT3) (Keep and Smith, 2011). Fluorescein, a known substrate for OAT3, is taken up by the transporter on the apical side of the choroid plexus epithelium in a Na^+^-dependent manner (Sykes et al., 2004). Because fluorescein is a low-molecular-weight green fluorescent dye, we soaked Casper zebrafish in 50 μM fluorescein to observe accumulation in the choroid plexus. The dye was ingested in the gut, entered the circulation, and concentrated in the diencephalic choroid plexus and myelencephalic choroid plexus (Figures 3G,H). Fluorescein injected into the brain ventricle had a similar pattern of accumulation in the myelencephalic choroid plexus and diencephalic choroid plexus (Figures 4E–H).

### Zebrafish choroid plexus possesses size-selective barrier properties

**Figure 5:**
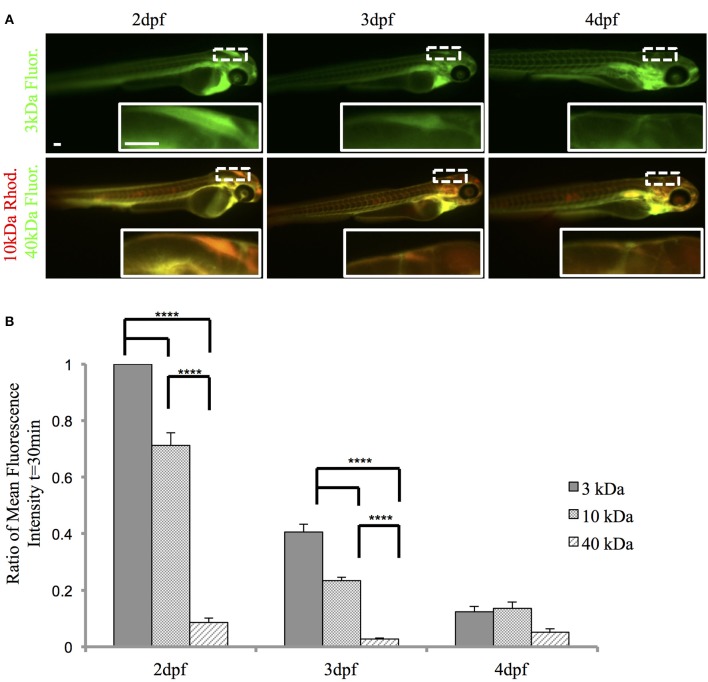
**The zebrafish CP is functional and possesses size-selective properties**. Casper zebrafish were intravenously injected with fluorescent tracers at 2, 3, and 4 dpf to identify tracer leakage from blood into the cerebrospinal fluid. **(A)** The top panel shows severe leakage of the 3-kDa fluorescein dextran into the brain ventricle (inset) at 2 dpf, minor leakage at 3 dpf, and little, if any, dextran penetration at 4 dpf. The bottom panel shows permeability of the 10-kDa rhodamine dextran at 2 dpf (inset), which decreases by 4 dpf. The 40-kDa fluorescein dextran was the least permeable at each developmental timepoint. Images were taken at 1 h post-injection. **(B)** A ratio of fluorescence intensity in the brain ventricle relative to the heart was measured at 30 min post-injection as a readout for tracer leakage into the brain ventricle. A One-Way ANOVA shows that the 3-kDa fluorescein dextran and the 10-kDa rhodamine dextran are significantly more permeable (i.e., greater mean fluorescence intensity) than the 40-kDa fluorescein dextran at 2 and 3 dpf. By 4 dpf, there was no significant difference in fluorescence intensity. Measurements are expressed as mean ± *SE* for *n* = 7; ^****^p < 0.0001. Abbreviations: Rhod, rhodamine dextran; Flour: fluorescein dextran. Scale bar is 50 μm.

**Figure 6:**
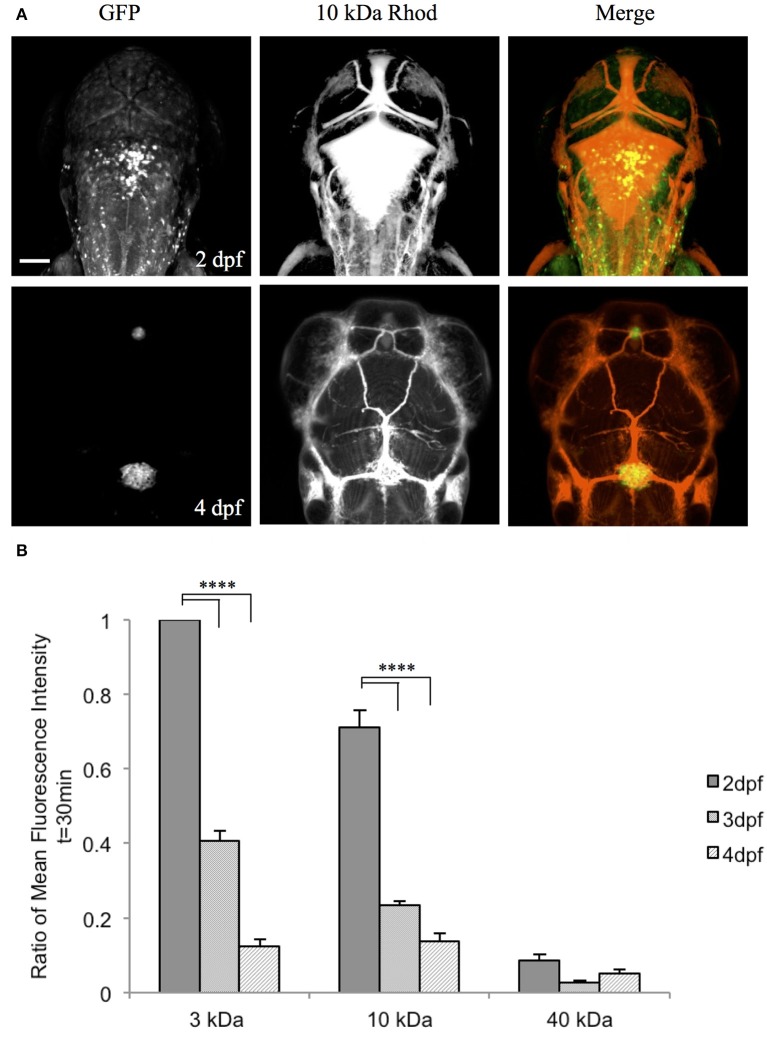
**The zebrafish CP becomes size-selective as it develops**. **(A)** *Et(cp:EGFP)*^*sj*2^ larvae were intravenously injected with a 10 kDa rhodamine-dextran and the CP was imaged dorsally by confocal microscopy. At 2 dpf, the CP is not fully developed as observed by GFP expression and the 10 kDa rhodamine-dextran inundates the brain ventricle. By 4 dpf, the CP is fully formed and the 10 kDa rhodamine-dextran is retained within the vasculature and does not enter the brain ventricle. **(B)** A One-Way ANOVA analysis revealed that the 3 kDa fluorescein dextran and 10 kDa rhodamine dextran have a significantly higher fluorescent intensity in the brain ventricle (i.e., more permeable) at 2 dpf compared to 3 and 4 dpf. There is no significant difference between developmental time points for the 40 kDa fluorescein dextran. Measurements are means ± *SE* for *n* = 7; ^****^p < 0.0001. Scale bar is 50 μm.

To examine the function of the zebrafish choroid plexus in regulating passage from the bloodstream into the cerebrospinal fluid, we intravenously injected fluorescently labeled dextrans of various molecular weights into the transparent Casper line at 2, 3, and 4 dpf. Time-lapse imaging at 2 dpf, before the choroid plexus has fully developed, showed that 3-kDa fluorescein and 10-kDa rhodamine dextrans leaked from the bloodstream into the brain ventricle, whereas little to no 40-kDa fluorescein dextran escaped from the bloodstream into the ventricle (Figure 5A). By 3 dpf, there was still a significant difference with a *P*-value of < 0.0001 between the 3- and 10-kDa dextrans and the 40-kDa dextran; however, the overall permeability was lower at 3 dpf compared to 2 dpf (Figure 5B). By 4 dpf, when the choroid plexus was fully formed, there were no significant differences between the individual tracers (Figure 5B). Dorsal views of an intravenous injection of the 10-kDa dextran into *Et(cp:EGFP)*^*sj*2^ larvae at 2 dpf showed leakage into the brain ventricle as the choroid plexus continued to develop. Once formed by 4 dpf, dextran did not accumulate within the brain ventricle (Figure 6A). In addition, the 3- and 10-kDa dextrans were significantly more permeable at 2 dpf (*P* < 0.0001) compared to 3 and 4 dpf, but there was no significant difference in permeability for the 40-kDa dextran (Figure 6B).

### Identification of choroid plexus mutants

After demonstrating that the zebrafish choroid plexus develops and functions as the blood-cerebrospinal fluid barrier beginning at 3 dpf, we initiated a small-scale forward genetic screen to identify mutants with choroid plexus abnormalities. This screen focused primarily on myelencephalic choroid plexus defects. Table 1 shows the numbers of fish generated from this screen. Briefly, 20 F_0_ *Et*(*cp:EGFP)*^*sj*2^ males were mutagenized with ENU. One month after treatment, ENU efficiency was tested by mating the males to Casper and screening for *nacre* or *roy* phenotypes. Approximately 1 in 250 (0.4%) larvae showed the *nacre* or *roy* phenotype. The mutagenized males were mated with *Et(cp:EGFP)*^*sj*2^ females to generate 409 F_1_ fish. Adult F_1_ pairs were mated together or with non-mutagenized *Et(cp:EGFP)*^*sj*2^ fish to produce 224 F_2_ families. Of the F_2_ families, 73 contained sufficient numbers to screen 4–6 pairs for mutants in the F_3_ generation. We set up 1330 F_2_ × F_2_ crosses (including re-crosses), with 421 crosses producing F_3_ embryos. Larvae were screened at 4 dpf to identify choroid plexus defects based on GFP expression patterns. In total, we generated approximately 16,339 F_3_ embryos and screened approximately 12,196.

**Table 1:** **Number of zebrafish generated from the forward genetic screen**.

**Generation**	**Fish (*n*)**
G_0_ males mutagenized	20
G_0_ males survived	9
F_1_	409
F_2_ families started	224
F_2_ families screened	73
F_2_ × F_2_ crosses	1330
F_2_ families that spawned	421
F_3_ embryos screened	12,196
Mutants lines identified	24
Mutant lines maintained	10

**Figure 7:**
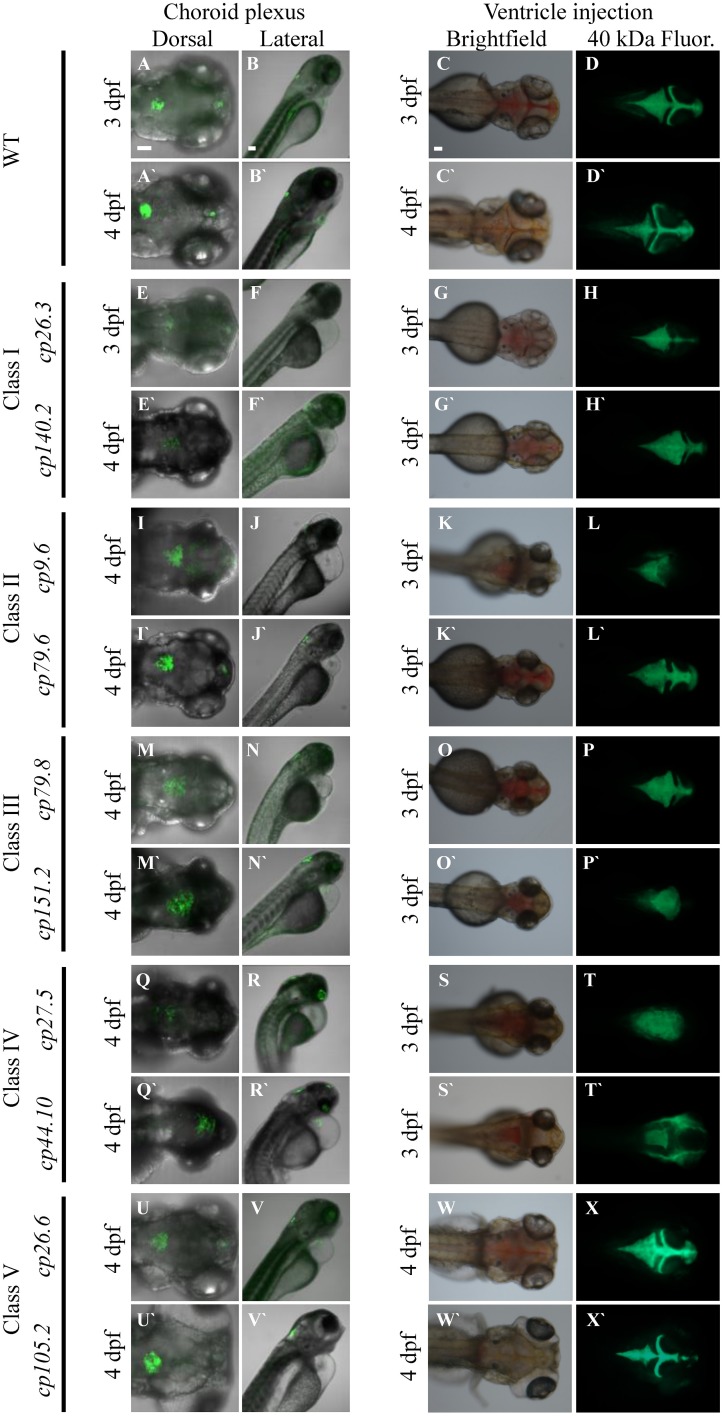
**Forward genetic screen identifies five classes of CP mutants**. **(A–B′)** Wild-type *Et(cp:EGFP)*^*sj*2^ larvae at 3 dpf and 4 dpf. **(C–C′)** Bright field image of 3 and 4 dpf wild-type after ventricle injection of the 40-kDa fluorescein dextran. **(D–D′)** Fluorescent image of 3 and 4 dpf wild-type after ventricle injection of the 40-kDa fluorescein dextran. Class I: **(E,F)** *cp26.3* at 3 dpf. **(E′,F′)** *cp140.2* at 4 dpf. **(G)** Bright field image of *cp26.3* at 3 dpf after ventricle injection of the 40-kDa fluorescein dextran. **(G′)** Bright field image of *cp140.2* at 3 dpf after ventricle injection of the 40-kDa fluorescein dextran. **(H)** Fluorescent image of *cp26.3* at 3 dpf after ventricle injection of the 40-kDa fluorescein dextran. **(H′)** Fluorescent image of *cp140.2* at 3 dpf after ventricle injection of the 40-kDa fluorescein dextran. Class II: **(I,J)** *cp9.6* at 4 dpf. **(I′,J′)** *cp79.6* at 4 dpf. **(K)** Bright field image of *cp9.6* at 3 dpf after ventricle injection of the 40-kDa fluorescein dextran. **(K′)** Bright field image of *cp79.6* at 3 dpf after ventricle injection of the 40-kDa fluorescein dextran. **(L)** Fluorescent image of *cp9.6* at 3 dpf after ventricle injection of the 40-kDa fluorescein dextran. **(L′)** Fluorescent image of *cp79.6* at 3 dpf after ventricle injection of the 40-kDa fluorescein dextran. Class III: **(M,N)** *cp79.8* at 4 dpf. **(M′,N′)** *cp151.2* at 4 dpf. **(O)** Bright field image of *cp79.8* at 3 dpf after ventricle injection of the 40-kDa fluorescein dextran. **(O′)** Bright field image of *cp151.2* at 3 dpf after ventricle injection of the 40-kDa fluorescein dextran. **(P)** Fluorescent image of *cp79.8* at 3 dpf after ventricle injection of the 40-kDa fluorescein dextran. **(P′)** Fluorescent image of *cp151.2* at 3 dpf after ventricle injection of the 40-kDa fluorescein dextran. Class IV: **(Q,R)** *cp27.5* at 4dpf. **(Q′,R′)** *cp44.10* at 4 dpf. **(S)** Bright field image of *cp27.5* at 3 dpf after ventricle injection of the 40-kDa fluorescein dextran. **(S′)** Bright field image of *cp44.10* at 3 dpf after ventricle injection of the 40-kDa fluorescein dextran. **(T)** Fluorescent image of *cp27.5* at 3 dpf after ventricle injection of the 40-kDa fluorescein dextran. **(T′)** Fluorescent image of *cp44.10* at 3 dpf after ventricle injection of the 40-kDa fluorescein dextran. Class V: **(U,V)** *cp26.6* at 4 dpf. **(U′,V′)** *cp105.2* at 4 dpf. **(W)** Bright field image of *cp26.6* at 4 dpf after ventricle injection of the 40-kDa fluorescein dextran. **(W′)** Bright field image of *cp105.2* at 4 dpf after ventricle injection of the 40-kDa fluorescein dextran. **(X)** Fluorescent image of *cp26.6* at 4 dpf after ventricle injection of the 40-kDa fluorescein dextran. **(X′)** Fluorescent image of *cp105.2* at 4 dpf after ventricle injection of the 40-kDa fluorescein dextran. The first, third, and fourth panels for each class are dorsal views while the second panel is a lateral view. The first and second panels for each class were acquired by confocal microscopy. The third and fourth panels were acquired by fluorescent stereoscopy. The first panel for each class is at 20× magnification, the second panel is at 10× magnification, and the third and fourth panels are at 16× magnification. 40 kDa Fluor, 40 kDa Fluorescein Dextran. Scale bars in A, B, and C are 50 μm.

We identified 24 mutant lines with 10 recessive mutant lines having choroid plexus deformities. Time-lapse imaging showing choroid plexus development for wild-type and some mutants are demonstrated in Supplemental Movies 2–7. In addition to choroid plexus deformities, each of the10 recessive lines had other defects such as heart edema, small eyes, and small head. While several of the mutants exhibited brain cell death, many did not develop cell death until after the choroid plexus started to form. A description of the onset of brain cell death for each line is listed in Table 2. We also found spontaneous mutants that had normal morphology overall, but severely abnormal or no choroid plexus (data not shown). In addition, we found many mutants (some recessive and some spontaneous) with severe brain cell death and normal choroid plexus development (data not shown). The choroid plexus mutant lines were named based on the original F_2_ family and pair number within that family. No mutants were adult viable and most did not survive past 4 dpf, except for *cp26.6* and *cp105.2*, which lived until 5 and 6 dpf, respectively. *cp27.5* and *cp44.10* mutants displayed similar phenotypes; however, complementation analysis showed that they were not in the same complementation group. On the basis of GFP intensity, epithelial localization, and overall choroid plexus morphology, we identified five classes: (I) mutants with reduced GFP expression, dispersed epithelial cells, and irregularly shaped choroid plexus; (II) mutants with normal GFP expression, small epithelial aggregates, and irregularly shaped choroid plexus; (III) mutants with normal GFP expression, small epithelial aggregates, and expanded choroid plexus; (IV) mutants with variable GFP expression, small epithelial aggregates, and expanded choroid plexus; and (V) mutants with normal GFP expression, compact epithelial cells, and enlarged choroid plexus. While complementation analysis was not performed between all of these mutants, we are fairly certain that these mutations are in different genes. However, we acknowledge that it is possible that different types of mutations in the same gene from different mutants could result in additional phenotypes. A description of these mutants and choroid plexus morphology are presented in Table 2 and Figure 7, respectively. Wild-type *Et(cp:EGFP)*^*sj*2^ larvae at 3 and 4 dpf are shown in Figures 7A–D′ as a reference to compare with mutant classes.

**Table 2:** **Characterization of *Et(cp:EGFP)* mutants generated from the forward genetic screen**.

**Class**	**Allele**	**Map position**	**DPF**	**Other phenotypes(4 dpf)**	**Fluorescein transport**	**Onset of brain cell death (up to 4 dpf)**	**Initial circulation loss (up to 4 dpf)**
I: reduced GFP expression, dispersed epithelia, and irregularly shaped CP	*cp26.3*^a^	ND	2–3	No swimbladder; brain and tail cell death; brain ventricle hemorrhaging; faint heartbeat; no circulation; no midbrain-hindbrain boundaries	Accumulates within third ventricle	3 dpf	3 dpf
	*cp140.2*	ND	2	Small head; small eyes; no swimbladder; heart edema; brain cell death; faint heartbeat and circulation; slightly larger third ventricle and wider lateral ventricles ventricle	Increased uptake; accumulates within brain ventricle	4 dpf	4 dpf
II: normal GFP expression, smaller epithelial aggregates, and irregularly shaped CP	*cp9.6*	LG 4	2	Small head; small eyes; no swimbladder; heart edema; occasional hydrocephalus; brain cell death; no circulation; no distinct third ventricle or lateral ventricles and severely reduced fourth ventricle	Increased uptake; accumulates within brain ventricle	2 dpf	4 dpf
	*cp79.6*	ND	2	Small head; small eyes; no swimbladder; heart edema; many still in chorion; brain cell death; hemorrhaging around heart; slow heartbeat; little to no circulation; wider lateral ventricles	Dispersed punctate staining on the dorsal ventricle surface	4 dpf	3 dpf
III: normal GFP expression, smaller epithelial aggregates, and expanded CP	*cp79.8*	ND	2	Small head; small eyes; no swimbladder; brain cell death; hemorrhaging around heart; decreased heartbeat and circulation; wider lateral ventricles	Increased uptake; accumulates within brain ventricle	4 dpf	3 dpf
	*cp151.2*	ND	2–3	Small head; small eyes; no swimbladder; brain cell death; minor swelling around head; occasional hemorrhaging around heart; smaller third ventricle and no defined lateral ventricles	Increased uptake; accumulates within brain ventricle	4 dpf	4 dpf
IV: variable GFP expression, smaller epithelial aggregates, and expanded CP	*cp27.5*	LG 21	2	Small head; small eyes; no swimbladder; brain cell death; heart edema; small arched body; curved tail; hydrocephalus; occasional hemorrhaging in brain ventricle; heartbeat, but little to no circulation; no defined ventricle boundaries	Increased uptake; accumulates within brain ventricle	2 dpf (localized to forebrain)	3 dpf
	*cp44.10*	ND	2	Small head; small eyes; no swimbladder; brain cell death; heart edema; small arched body; curved tail; hydrocephalus; occasional hemorrhaging around heart; heartbeat, but little to no circulation; no third ventricle or lateral ventricles and irregularly shaped fourth ventricle	Increased uptake; accumulates within brain ventricle	2 dpf (localized to forebrain)	4 dpf
V: normal GFP expression, compact epithelia, and enlarged CP	*cp26.6*^b^	ND	4	Rounded head, protruding lower jaw; no swimbladder; edema around heart, eyes, and gut; faint heartbeat and circulation; normal brain ventricle	N/A	N/A	4 dpf
	*cp105.2*^b^	ND	4–5	Rounded head, protruding lower jaw; no swimbladder; severe edema around heart, eyes, and gut; faint heartbeat and circulation; slightly smaller fourth ventricle	N/A	N/A	6 dpf

a Observations recorded at 3 dpf;

b observations recorded at 5 dpf;

ND, not determined; N/A, not applicable.

To further classify these mutants, we analyzed brain ventricle morphology and transporter activity in the choroid plexus. To examine brain ventricle morphology, we injected a 40-kDa fluorescein dextran into the ventricle of *Et(cp:EGFP)*^*sj*2^ mutants that were generated by outcrossing the mutant lines to the wild-type TL strain, were GFP negative, and selected based upon phenotypic traits. Descriptions of brain ventricles and their morphology are presented in Table 2 and Figure 7, respectively. None of the mutants except *cp26.6* and *cp105.2* had normal ventricle morphology, While some mutants such as *cp79.6* and *cp79.8* had a third, fourth, and lateral ventricles, the shape or size of the ventricle differed from wild-type. None of the mutants except *cp26.6* and *cp105.2* had normal ventricle morphology. Severe cases having no defined ventricular boundaries were observed in mutants *cp9.6, cp27.5*, *cp44.10*, and *cp151.2*.

### Fluorescein assay to determine choroid plexus transport

**Figure 8:**
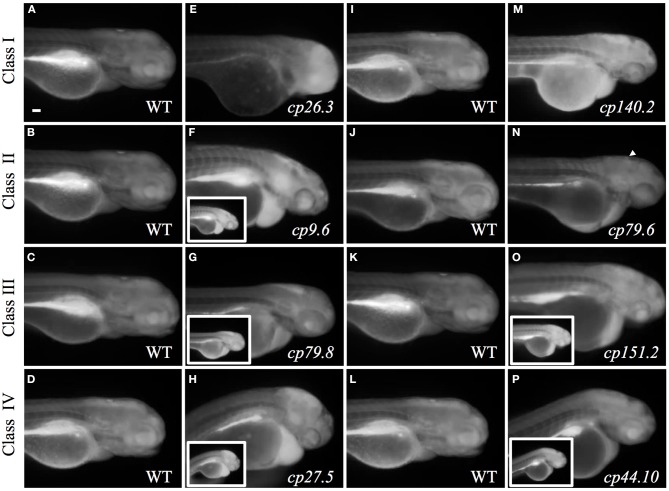
**Fluorescein assay determines CP transport activity in mutants**. **(A–D)** and **(I–L)** Wild-type larvae treated at 3 dpf with 50 μM fluorescein. **(E)** *cp26.3* mutant at 3 dpf. **(F)** *cp9.6* mutant at 3 dpf. Inset image is acquired at the same exposure to the corresponding wild-type in **(B)**. **(G)** *cp79.8* mutant at 3 dpf. Inset image is acquired at the same exposure to the corresponding wild-type in **(C)**. **(H)** *cp27.5* mutant at 3 dpf. Inset image is acquired at the same exposure to the corresponding wild-type in **(D)**. **(M)** *cp140.2* mutant at 3 dpf. **(N)** *cp79.6* mutant at 3 dpf. Punctate accumulation of fluorescein in the brain ventricle is shown with an arrowhead. **(O)** *cp151.2* mutant at 3 dpf. Inset image is acquired at the same exposure to the corresponding wild-type in **(K)**. **(P)** *cp44.10* mutant at 3 dpf. Inset image is acquired at the same exposure to the corresponding wild-type in **(L)**. The larger images for **(F**,**G**,**H**,**O**,**P)** were taken at a lower exposure compared to the corresponding wild-type in order to visualize where the fluorescein was concentrating throughout the larvae. Scale bar is 50 μm.

Fluorescein transport was used as a secondary assay to measure transporter activity in the choroid plexus and overall permeability (refer to Figures 3G,H). To determine if the choroid plexus could transport fluorescein in the mutants, larvae were treated at 3 dpf with 50 μM fluorescein as described above (Figure 8). In Class I mutants, *cp26.3* had fluorescein accumulation toward the anterior part of the brain and no fluorescein accumulated within the choroid plexus (Figure 8E). Fluorescein did not accumulate in the choroid plexus epithelium in the *cp140.2* mutant, but was localized throughout the brain ventricle. These mutants also had a higher overall fluorescein uptake than wildtype. (Figure 8M).

In Class II mutants, *cp9.6* mutants also exhibited a higher overall uptake of fluorescein than wild-type and concentrated within the brain ventricle, gut, and heart. There was no distinct accumulation within the choroid plexus (Figure 8F). For *cp79.6*, the overall uptake of fluorescein was comparable to wild-type. We observed very faint punctate accumulate of fluorescein within the general area of the choroid plexus indicating possible transport (Figure 8N).

Class III mutants (*cp79.8* and *cp151.2*) had increased uptake of fluorescein compared to wild-type and both had accumulation within the brain ventricle, gut, and heart. No fluorescein accumulated within the choroid plexus epithelium (Figures 8G–O). A similar phenotype was observed in Class IV mutants (*cp27.5* and *cp44.10*) in that they also had increased uptake of fluorescein concentrated within the brain ventricle, gut, and heart, but no localization within choroid plexus epithelium (Figures 8H–P). We were not able conclude whether Class V mutants (*cp26.6* and *cp105.2*) demonstrated transport activity using this assay because of the inability to confidently identify GFP negative mutants at 3 and 4 dpf. Once we identified mutants by late 4–5 dpf, the fluorescein was rapidly cleared by the gut and was not distributed throughout the embryo (data not shown).

### Genetic mapping of *cp9.6* and *cp27.5*

To identify the mutated gene for lines *cp9.6* and *cp27.5*, which displayed hydrocephaly phenotypes, we performed genetic mapping using 192 polymorphic markers distributed evenly across the genome. By bulked segregant analysis, we found linkage to marker z3275 that mapped *cp9.6* to chromosome 4. With z3275, we tested 100 mutants and identified 4 recombinants in 200 meiotic events. The critical interval along the chromosome was defined by recombination analysis. We identified polymorphic markers on either side of z3275, z23802, and z15751; however, the lack of additional polymorphic markers within this region made it difficult to define a reasonable mapping distance.

For *cp27.5*, we identified markers z2363 and z15891 by bulked segregant analysis to map the mutation to chromosome 21. Within this critical interval, we identified the marker z9233 and found no recombinants out of 48 meioses. We then tested additional polymorphic markers and counted the number of recombinants on either side of z9233 to refine the critical interval. By testing polymorphic markers, BX530031.5 and BX530023.5, that we selected from the zebrafish genome published in Ensembl Zv9, we narrowed the region to ~0.4 Mb. In total, we analyzed 450 meioses. Future analysis will include testing candidate genes within this interval to identify the mutated gene.

## Discussion

The goal of our study was to establish zebrafish as a functionally relevant model to study choroid plexus development and as a screening tool to genetically dissect genes involved in choroid plexus formation, function, and maintenance. Although the choroid plexus has been studied in various *in vivo* and *in vitro* models (Dohrmann, 1970; Strazielle and Preston, 2003), the developmental and genetic aspects of this structure remain poorly understood largely due to the lack of innovative strategies and functionally relevant systems (Saunders et al., 2008). Also, current models do not easily allow for choroid plexus development to be visualized in real time. To overcome this limitation, we created an enhancer trap zebrafish line, *Et*(*cp:EGFP*)^*sj*2^, which expresses EGFP within the diencephalic choroid plexus and myelencephalic choroid plexus. Using this line, choroid plexus development was easily observed in a live vertebrate by monitoring EGFP expression.

Previous studies have also generated enhancer trap lines in zebrafish expressing GFP in the choroid plexus (Bill et al., 2008; Garcia-Lecea et al., 2008). However, there are variations within these lines, including the subsets of cells expressing GFP. For example, in the *Et*^*Mn*16^ line (Bill et al., 2008), only a subset of cells of the myelencephalic choroid plexus express GFP. In addition, the *SqET33-E20* (Gateways) line has additional GFP expression in the rhombomeres (Garcia-Lecea et al., 2008). In our study, there was specific GFP expression in the diencephalic choroid plexus, myelencephalic choroid plexus, and swimbladder. In regards to GFP expression in the swimbladder, studies have shown that Sonic hedgehog (Shh) is expressed in the epithelial cells lining the swimbladder (Winata et al., 2009). Interestingly, the choroid plexus of other vertebrates has also been compared to the mammalian lung in a study by Nielsen and Dymecki (2010) demonstrating that Shh (also expressed in choroid plexus epithelia) is required for the synchronized outgrowth of the choroid plexus and fenestrated vasculature, similar to the need for Shh in coordinating the outgrowth of the lung (Nielsen and Dymecki, 2010). Since the zebrafish swimbladder has been compared to the mammalian lung (Winata et al., 2009), common underlying developmental pathways, such as Shh, required in both choroid plexus and swimbladder development, maybe be regulating GFP expression in these tissues. The variations in other enhancer trap lines may be due to transgenic DNA constructs integrating into different regions of the genome. Our PCR and Southern blot analysis revealed a single integration on chromosome 13. In contrast, the Gateways line has a single insertion on chromosome 24 (Garcia-Lecea et al., 2008), which likely accounts for the differences in expression patterns between lines. Similar to the previously published zebrafish choroid plexus papers (Bill et al., 2008; Garcia-Lecea et al., 2008), we were unable to identify the enhancer regulating GFP expression in the choroid plexus. This is often true with enhancer trap effects where is difficult to identify the enhancer if it acts at distances upstream or downstream of the reporter gene.

We have demonstrated blood-cerebrospinal fluid barrier properties in zebrafish by identifying tight junction proteins and transporter activity. We have shown Cldn5 expression within the choroid plexus and demonstrated that the zebrafish choroid plexus possess a chemical barrier that transports and concentrates rhodamine 123 and fluorescein in the choroid plexus epithelium. Rhodamine 123 directly interacts with MRP1 and MDR1 in an ATP-dependent manner (Shapiro and Ling, 1998; Daoud et al., 2000). Because previous studies have shown the specificity of rhodamine 123 to multidrug resistance proteins (MDRs), similar drug transporters are likely expressed in the zebrafish choroid plexus. Studies in mice have identified known transporters of rhodamine 123 such as MRP1 to be localized at the basolateral side of choroid plexus epithelium and have been shown to keep drugs such as etoposide out of the cerebrospinal fluid (Wijnholds et al., 2000; Keep and Smith, 2011). Our findings also confirm that transporters are present in the zebrafish choroid plexus by showing that: when coming from the bloodstream, rhodamine 123 accumulates within the choroid plexus and is prevented from entering the cerebrospinal fluid. When injected directly into the cerebrospinal fluid, the choroid plexus removed rhodamine 123 from the fluid as shown by dye accumulation within choroid plexus epithelium. Unfortunately, due to the lack of antibodies that cross-react with zebrafish proteins, we were unable to demonstrate the expression of specific transporters in the zebrafish choroid plexus. We tested an antibody against MDR1 (C219), but found expression only within brain endothelial cells of the blood-brain barrier (Umans and Taylor, 2012) and not the choroid plexus (data not shown). We also tested several commercially available antibodies against OAT3, but none cross-reacted with zebrafish. Additionally, while we did not observe Glut1 expression within the zebrafish choroid plexus, a recent review by Keep and Smith indicated that the BBB, not the blood-cerebrospinal fluid barrier, may be the major source of transporting glucose into the brain and that the choroid plexus may play a minor role in glucose transport (Keep and Smith, 2011).

We demonstrated barrier function in the zebrafish choroid plexus by injecting fluorescent tracers of different molecular weights and observing permeability across the choroid plexus epithelium into the cerebrospinal fluid. Once the choroid plexus fully formed by 4 dpf, the smallest tracer tested, a 3-kDa fluorescein dextran, was no longer permeable across the blood-cerebrospinal fluid barrier as it was in earlier developmental stages. This result indicated a tightening of the barrier as it develops. Although we did not distinguish between transcellular and paracellular transport, there appears to be a correlation between choroid plexus formation and decreased permeability, suggesting that a physical barrier is preventing molecule entry rather than being cleared by a cerebrospinal fluid drainage pathway. For example, this is demonstrated when we injected the 40-kDa tracer into the bloodstream on day 4 and never observed tracer accumulation within the ventricle throughout the course of the timelapse. Rather than suggesting that the tracer is being cleared from the ventricle by cerebrospinal fluid flow, we observed that the tracer never entered the ventricle in the first place. This indicated to us that a physical barrier is preventing the dextran from crossing the barrier. Had we observed accumulation of the dextran within the cerebrospinal fluid/brain ventricle after injection, and then observed a decrease in the amount of tracer in the ventricle over time, then we would suggest that cerebrospinal fluid flow is the contributing factor and not a physical blockage of the dextran by the barrier as we observed. In immature brain barriers, it has been reported that the accumulation of molecules within the cerebrospinal fluid is not due to increased permeability of the barrier but reduced cerebrospinal fluid flow (Saunders et al., 2008). In contrast, more mature brain barriers have increased cerebrospinal fluid flow that limits the accumulation of molecules within the ventricular space (Saunders et al., 2008). Our study showed that after the formation of the choroid plexus there was no tracer penetration into the ventricle. Therefore, a decrease in tracer accumulation in the brain ventricle does not appear to be due to increased cerebrospinal fluid clearance, but due to physical barrier properties of the choroid plexus. The decrease in barrier permeability as the choroid plexus develops establishes the zebrafish choroid plexus as a functioning organ with size-selective barrier properties.

Forward genetic screens have been performed in various multicellular organisms such as plants (Meinke and Sussex, 1979), flies (Nusslein-Volhard and Wieschaus, 1980), worms (Sin et al., 2014), zebrafish (Driever et al., 1996), and mice (Nolan et al., 2000). These screens have been very successful in discovering genes involved in embryonic patterning, organ development, and behavior (Stemple et al., 1996; Horvitz, 1999; Muto et al., 2005). To characterize the mutants in our study, we examined not only GFP expression in the choroid plexus but also ventricle morphology and transporter activity. Previous studies have grouped zebrafish brain ventricle mutants into four categories: midline separation defects, reduced ventricle size, lateral ventricles and fourth ventricle abnormalities, and absence of lumen inflation (Lowery et al., 2009). Because none of our choroid plexus mutants exhibited a phenotype at 24 hpf, we did not expect to see defects in midline separation as described in Lowery et al. (2009). Choroid plexus mutants such as *cp79.6* and *cp140.2* have a brain ventricle that closely resembles normal larvae at 2 dpf (Lowery et al., 2007), indicating these mutants may have a developmental delay and not necessarily a brain ventricle defect. Also, *cp140.2* mutants at 3 dpf are similar to wild-type larvae at 2 dpf in that the fluorescein accumulated within the brain ventricle rather than concentrating within choroid plexus epithelium as it does normally on day 3. Many mutants exhibited an increased overall uptake of fluorescein, which may indicate defects in the skin epithelial barrier as well and increased permeability to absorb small molecules from their surroundings.

While mutants exhibit little to no heart beat and circulation when screened on day 4, as described in Table 2, the majority of mutants do have a strong heart beat and rapid circulation indistinguishable from wild-type prior to this timepoint. The initial onset of circulation loss describes when we first observed mutants with decreased or no circulation. Not all of the mutants had loss of circulation at the same time, but the onset describes when we observed the first mutant from a spawn having no circulation. Because most began to have decreased heartbeat and circulation at 4 dpf after the choroid plexus has already developed, we conclude that other factors are regulating choroid plexus development. However, we cannot rule out that circulation is required for choroid plexus maintenance. We also do not attribute the lack of blood flow to abnormal choroid plexus development, because we identified spontaneous mutants with a normal choroid plexus but no circulation (data not shown). After injecting single-cell embryos with a cardiac troponin morpholino to block heartbeat (Sehnert et al., 2002), we identified no choroid plexus defects resembling abnormalities of the five mutant classes (data not shown). Therefore, we conclude that if loss of circulation alone was the factor governing abnormal choroid plexus development in these mutants, we should see a choroid plexus that resembled that of troponin morphants. Because many mutants continued to have a heartbeat and circulation at 3 dpf, which is essential for brain ventricle expansion (Lowery and Sive, 2005), we also did not expect ventricle expansion defects in these mutants. While ventricle morphology varied between mutants, all had expanded ventricles.

The mutants identified in our screen will provide the tools necessary to investigate several genes associated with choroid plexus development and barrier function. After isolating the defective genes from these mutants, we can define the correlation between the genes involved and the phenotypic abnormalities we observed. Although we identified mutants with brain cell death, we do not contribute cell death alone as a factor responsible for abnormal choroid plexus development and believe that the processes can be unrelated. This is supported by the identification of larvae with brain cell death developed a normal choroid plexus. Furthermore, for the mutants we characterized further, we did not observe the onset of cell death until after the choroid plexus had already started to develop for many of the lines, or if cell death was present, it was not near the choroid plexus itself. We also found larvae that had normal morphology overall, but abnormal or no choroid plexus indicating again that cell death is not the sole mechanism responsible for abnormal choroid plexus development and that specific pathways exist in regulation choroid plexus formation and function.

We expect some of the genes we identify will be specifically related to a choroid plexus defect rather than housekeeping genes causing an overall severely abnormal animal. Recent discoveries have shed light on the importance of the choroid plexus and its contribution to disease. For example, transcriptome analysis during early development revealed that the choroid plexus expresses numerous genes essential for choroid plexus function, including those for tight junctions, transporters, and metabolic function (Liddelow et al., 2012), protecting the brain from potentially harmful insults (Kratzer et al., 2013). Previous studies have also identified genetic components associated with choroid plexus dysfunction and disease. Genes involved in these pathologies include Polycystin-1 deficiency, which contributes to hydrocephaly (Wodarczyk et al., 2009). Because some types of hydrocephalus can be congenital, we plan to look at the mutant lines displaying hydrocephalus-like phenotypes with expanded ventricles (e.g., *cp27.5* and *cp9.6*) and determine whether we can find genetic linkage to this phenotype. In addition, mutations in Twist-related protein-1 and constitutively active Notch 3 promote proliferation and invasion in choroid plexus tumors (Wolburg and Paulus, 2010). Furthermore, disruption of the blood-cerebrospinal fluid barrier in multiple sclerosis can cause inflammation and leakage of T lymphocytes into the cerebrospinal fluid (Vercellino et al., 2008; Wolburg and Paulus, 2010). In patients with Alzheimer's disease, the choroid plexus has decreased function in amyloid beta plaque clearance (Alvira-Botero and Carro, 2010; Wolburg and Paulus, 2010). Since the mutations in the choroid plexus lines we identified are embryonic lethal, they may not be good representations to model later life diseases such as multiple sclerosis or Alzheimer's disease. However, we can investigate whether the choroid plexus is compromised in these mutants in regards to specific aspects of these diseases, such as an increased influx of immune cells into the cerebrospinal fluid characterized in multiple sclerosis, or reduced cerebrospinal fluid flow found in Alzheimer's disease patients. Identifying the genetic mutations that produce similar pathologies of these diseases may provide insight into the mechanisms that occur in the choroid plexus at later stages in life for these conditions.

The choroid plexus mutants identified in our study will be used to identify genes essential for maintaining epithelial barrier integrity as well as genes essential for initial barrier formation. Reverse genetic studies have shown that transcription factors such as *Otx2* aid in choroid plexus development and maintenance (Johansson et al., 2013). Also, augurin, a protein encoded by the esophageal cancer related gene-4 (*Ecrg4*) and thought to act as a tumor suppressor, has a higher expression in the choroid plexus than other tissues. Interestingly, morpholino knockdown of *Ecrg4* in zebrafish results in increased central nervous system cell proliferation and hydrocephaly (Gonzalez et al., 2011). Additional studies in zebrafish showed that the clusterin gene, which encodes a secreted glycoprotein that aids in aggregation and contributes to Alzheimer's disease by colocalizing with plaques (Thambisetty et al., 2010), is similar to its human counterpart and is expressed in the diencephalic choroid plexus and myelencephalic choroid plexus (Jiao et al., 2011). Demonstrating the similarity in gene expression in zebrafish and human choroid plexus is important when considering the use of zebrafish as a disease model for choroid plexus-related conditions. Our study provides evidence that the barrier properties and function of zebrafish choroid plexus are similar to those of mammalian choroid plexus. Ultimately, the genes discovered from our choroid plexus mutants will provide a better understanding of the genetic and molecular mechanisms involved in choroid plexus formation and function and new insights into how these normal processes become altered in disease.

## Author contributions

Hannah E. Henson wrote the manuscript and Michael R. Taylor edited the manuscript. Hannah E. Henson performed the characterization study including Claudin 5 staining, transporter experiments, and fluorescent tracer experiments. Hannah E. Henson also performed the forward genetic screen, fluorescein assay, and genetic mapping. Chaithanyarani Parupalli performed the forward genetic screen. Chaithanyarani Parupalli and Hannah E. Henson performed the time-lapse imaging on wild-type and choroid plexus mutants. Bensheng Ju and Michael R. Taylor generated the *Et(cp:EGFP)*^*sj*2^ line.

### Conflict of interest statement

The authors declare that the research was conducted in the absence of any commercial or financial relationships that could be construed as a potential conflict of interest.

## Abbreviations

Cldn5: Claudin 5
CNS: central nervous system
CP: choroid plexus
CPe: choroid plexus epithelium
CSF: cerebrospinal fluid
dpf: days post-fertilization
dCP: diencephalic choroid plexus
EGFP: enhanced green fluorescent protein
*Et*(*cp:EGFP*)^*sj*2^: *Enhancer trap*(*choroid plexus:enhanced green fluorescent protein*)^*sj*2^
hpf: hours post-fertilization
mCP: myelencephalic choroid plexus
MDR1: Multidrug Resistance Transporter 1
MRP1: Multidrug Resistance-Associated Protein 1
OAT3: Organic Anion Transporter 3
PTU: N-Phenylthiourea.

## References

[B1] Alvira-Botero X. Carro E. M. (2010). Clearance of amyloid-beta peptide across the choroid plexus in Alzheimer's disease. Curr. Aging Sci. 3, 219–229. 10.2174/1874609811003030219 20735345

[B2] Bill B. R. Balciunas D. McCarra J. A. Young E. D. Xiong T. Spahn A. M.. (2008). Development and Notch signaling requirements of the zebrafish choroid plexus. PLoS ONE 3:e3114. 10.1371/journal.pone.0003114 18769591 PMC2528000

[B3] Craig M. P. Gilday S. D. Dabiri D. Hove J. R. (2012). An optimized method for delivering flow tracer particles to intravital fluid environments in the developing zebrafish. Zebrafish 9, 108–119. 10.1089/zeb.2012.0740 22985309 PMC3444766

[B4] Currle D. S. Cheng X. Hsu C. M. Monuki E. S. (2005). Direct and indirect roles of CNS dorsal midline cells in choroid plexus epithelia formation. Development 132, 3549–3559. 10.1242/dev.01915 15975937

[B5] Daoud R. Kast C. Gros P. Georges E. (2000). Rhodamine 123 binds to multiple sites in the multidrug resistance protein (MRP1). Biochemistry 39, 15344–15352. 10.1021/bi0020574 11112520

[B6] Dohrmann G. J. (1970). The choroid plexus: a historical review. Brain Res. 18, 197–218. 10.1016/0006-8993(70)90324-0 4929003

[B7] Driever W. Solnica-Krezel L. Schier A. F. Neuhauss S. C. Malicki J. Stemple D. L.. (1996). A genetic screen for mutations affecting embryogenesis in zebrafish. Development 123, 37–46. 9007227 10.1242/dev.123.1.37

[B8] Dziegielewska K. M. Ek J. Habgood M. D. Saunders N. R. (2001). Development of the choroid plexus. Microsc. Res. Tech. 52, 5–20. 10.1002/1097-0029(20010101)52:1<5::AID-JEMT3>3.0.CO;2-J 11135444

[B9] Folgueira M. Bayley P. Navratilova P. Becker T. S. Wilson S. W. Clarke J. D. (2012). Morphogenesis underlying the development of the everted teleost telencephalon. Neural Dev. 7, 32. 10.1186/1749-8104-7-32 22989074 PMC3520737

[B10] Garcia-Lecea M. Kondrychyn I. Fong S. H. Ye Z. R. Korzh V. (2008). *In vivo* analysis of choroid plexus morphogenesis in zebrafish. PLoS ONE 3:e3090. 10.1371/journal.pone.0003090 18769618 PMC2525818

[B11] Goldsmith J. R. Jobin C. (2012). Think small: zebrafish as a model system of human pathology. J. Biomed. Biotechnol. 2012:817341. 10.1155/2012/817341 22701308 PMC3371824

[B12] Gonzalez A. M. Podvin S. Lin S. Y. Miller M. C. Botfield H. Leadbeater W. E.. (2011). Ecrg4 expression and its product augurin in the choroid plexus: impact on fetal brain development, cerebrospinal fluid homeostasis and neuroprogenitor cell response to CNS injury. Fluids Barriers CNS 8:6. 10.1186/2045-8118-8-6 21349154 PMC3042980

[B13] Gutzman J. H. Sive H. (2009). Zebrafish brain ventricle injection. J. Vis. Exp. 26:e1218. 10.3791/1218 19352312 PMC2791086

[B14] Horvitz H. R. (1999). Genetic control of programmed cell death in the nematode Caenorhabditis elegans. Cancer Res. 59, 1701s-1706s. 10197583

[B15] Huang X. Ketova T. Fleming J. T. Wang H. Dey S. K. Litingtung Y.. (2009). Sonic hedgehog signaling regulates a novel epithelial progenitor domain of the hindbrain choroid plexus. Development 136, 2535–2543. 10.1242/dev.033795 19570847 PMC2709062

[B16] Irvin D. K. Nakano I. Paucar A. Kornblum H. I. (2004). Patterns of Jagged1, Jagged2, Delta-like 1 and Delta-like 3 expression during late embryonic and postnatal brain development suggest multiple functional roles in progenitors and differentiated cells. J. Neurosci. Res. 75, 330–343. 10.1002/jnr.10843 14743446

[B17] Jansen W. F. De Weger R. A. Woutersen R. A. Van Loveren H. Van De Kamer J. C. (1976). The saccus dorsalis of the rainbow trout, Salmo gairdneri Richardson: histological, cytochemical, electron microscopical and autoradiographical observations. Cell Tissue Res. 167, 467–491. 10.1007/BF00215179 131647

[B18] Janssen S. F. Van Der Spek S. J. Ten Brink J. B. Essing A. H. Gorgels T. G. Van Der Spek P. J.. (2013). Gene expression and functional annotation of the human and mouse choroid plexus epithelium. PLoS ONE 8:e83345. 10.1371/journal.pone.0083345 24391755 PMC3877019

[B19] Jiao S. Dai W. Lu L. Liu Y. Zhou J. Li Y.. (2011). The conserved clusterin gene is expressed in the developing choroid plexus under the regulation of notch but not IGF signaling in zebrafish. Endocrinology 152, 1860–1871. 10.1210/en.2010-1183 21385939

[B20] Johansson P. A. Irmler M. Acampora D. Beckers J. Simeone A. Gotz M. (2013). The transcription factor Otx2 regulates choroid plexus development and function. Development 140, 1055–1066. 10.1242/dev.090860 23364326

[B21] Keep R. F. Smith D. E. (2011). Choroid plexus transport: gene deletion studies. Fluids Barriers CNS 8:26. 10.1186/2045-8118-8-26 22053861 PMC3231976

[B22] Kratzer I. Liddelow S. A. Saunders N. R. Dziegielewska K. M. Strazielle N. Ghersi-Egea J. F. (2013). Developmental changes in the transcriptome of the rat choroid plexus in relation to neuroprotection. Fluids Barriers CNS 10:25. 10.1186/2045-8118-10-25 23915922 PMC3737068

[B23] Kratzer I. Vasiljevic A. Rey C. Fevre-Montange M. Saunders N. Strazielle N.. (2012). Complexity and developmental changes in the expression pattern of claudins at the blood-CSF barrier. Histochem. Cell Biol. 138, 861–879. 10.1007/s00418-012-1001-9 22886143 PMC3483103

[B24] Lagaraine C. Skipor J. Szczepkowska A. Dufourny L. Thiery J. C. (2011). Tight junction proteins vary in the choroid plexus of ewes according to photoperiod. Brain Res. 1393, 44–51. 10.1016/j.brainres.2011.04.009 21529785

[B25] Liddelow S. A. Dziegielewska K. M. Mollgard K. Whish S. C. Noor N. M. Wheaton B. J.. (2014). Cellular Specificity of the Blood-CSF barrier for albumin transfer across the choroid plexus epithelium. PLoS ONE 9:e106592. 10.1371/journal.pone.0106592 25211495 PMC4161337

[B26] Liddelow S. A. Dziegielewska K. M. Vandeberg J. L. Saunders N. R. (2010). Development of the lateral ventricular choroid plexus in a marsupial, monodelphis domestica. Cerebrospinal Fluid Res. 7:16. 10.1186/1743-8454-7-16 20920364 PMC2964622

[B27] Liddelow S. A. Temple S. Mollgard K. Gehwolf R. Wagner A. Bauer H.. (2012). Molecular characterisation of transport mechanisms at the developing mouse blood-CSF interface: a transcriptome approach. PLoS ONE 7:e33554. 10.1371/annotation/2a9099a5-688b-4def-95a7-6ac13b10d096 22457777 PMC3310074

[B28] Lippoldt A. Liebner S. Andbjer B. Kalbacher H. Wolburg H. Haller H.. (2000). Organization of choroid plexus epithelial and endothelial cell tight junctions and regulation of claudin-1, -2 and -5 expression by protein kinase C. Neuroreport 11, 1427–1431. 10.1097/00001756-200005150-00015 10841351

[B29] Lowery L. A. De Rienzo G. Gutzman J. H. Sive H. (2009). Characterization and classification of zebrafish brain morphology mutants. Anat. Rec. (Hoboken) 292, 94–106. 10.1002/ar.20768 19051268 PMC2894611

[B30] Lowery L. A. Rubin J. Sive H. (2007). Whitesnake/sfpq is required for cell survival and neuronal development in the zebrafish. Dev. Dyn. 236, 1347–1357. 10.1002/dvdy.21132 17393485

[B31] Lowery L. A. Sive H. (2005). Initial formation of zebrafish brain ventricles occurs independently of circulation and requires the nagie oko and snakehead/atp1a1a.1 gene products. Development 132, 2057–2067. 10.1242/dev.01791 15788456

[B32] Meinke D. W. Sussex I. M. (1979). Embryo-lethal mutants of Arabidopsis thaliana. A model system for genetic analysis of plant embryo development. Dev. Biol. 72, 50–61. 10.1016/0012-1606(79)90097-6 510780

[B33] Monnot A. D. Zheng W. (2013). Culture of choroid plexus epithelial cells and *in vitro* model of blood-CSF barrier. Methods Mol. Biol. 945, 13–29. 10.1007/978-1-62703-125-7_2 23097098 PMC3982224

[B34] Muto A. Orger M. B. Wehman A. M. Smear M. C. Kay J. N. Page-Mccaw P. S.. (2005). Forward genetic analysis of visual behavior in zebrafish. PLoS Genet. 1:e66. 10.1371/journal.pgen.0010066 16311625 PMC1287954

[B35] Nielsen C. M. Dymecki S. M. (2010). Sonic hedgehog is required for vascular outgrowth in the hindbrain choroid plexus. Dev. Biol. 340, 430–437. 10.1016/j.ydbio.2010.01.032 20123094 PMC2897143

[B36] Nolan P. M. Peters J. Strivens M. Rogers D. Hagan J. Spurr N.. (2000). A systematic, genome-wide, phenotype-driven mutagenesis programme for gene function studies in the mouse. Nat. Genet. 25, 440–443. 10.1038/78140 10932191

[B37] Nusslein-Volhard C. Wieschaus E. (1980). Mutations affecting segment number and polarity in Drosophila. Nature 287, 795–801. 10.1038/287795a0 6776413

[B38] Omura Y. Korf H. W. Oksche A. (1985). Vascular permeability (problem of the blood-brain barrier) in the pineal organ of the rainbow trout, Salmo gairdneri. Cell Tissue Res. 239, 599–610. 10.1007/BF00219238 2580630

[B39] Saengkhae C. Loetchutinat C. Garnier-Suillerot A. (2003). Kinetic analysis of rhodamines efflux mediated by the multidrug resistance protein (MRP1). Biophys. J. 85, 2006–2014. 10.1016/S0006-3495(03)74628-1 12944313 PMC1303372

[B40] Saunders N. R. Ek C. J. Habgood M. D. Dziegielewska K. M. (2008). Barriers in the brain: a renaissance? Trends Neurosci. 31, 279–286. 10.1016/j.tins.2008.03.003 18471905

[B41] Schmidt R. Strahle U. Scholpp S. (2013). Neurogenesis in zebrafish - from embryo to adult. Neural Dev. 8:3. 10.1186/1749-8104-8-3 23433260 PMC3598338

[B42] Sehnert A. J. Huq A. Weinstein B. M. Walker C. Fishman M. Stainier D. Y. (2002). Cardiac troponin T is essential in sarcomere assembly and cardiac contractility. Nat. Genet. 31, 106–110. 10.1038/ng875 11967535

[B43] Shapiro A. B. Ling V. (1998). Stoichiometry of coupling of rhodamine 123 transport to ATP hydrolysis by P-glycoprotein. Eur. J. Biochem. 254, 189–193. 10.1046/j.1432-1327.1998.2540189.x 9652413

[B44] Sin O. Michels H. Nollen E. A. (2014). Genetic screens in Caenorhabditis elegans models for neurodegenerative diseases. Biochim. Biophys. Acta 1842, 1951–1959. 10.1016/j.bbadis.2014.01.015 24525026

[B45] Skinner D. C. Malpaux B. (1999). High melatonin concentrations in third ventricular cerebrospinal fluid are not due to Galen vein blood recirculating through the choroid plexus. Endocrinology 140, 4399–4405. 10499491 10.1210/endo.140.10.7074

[B46] Stemple D. L. Solnica-Krezel L. Zwartkruis F. Neuhauss S. C. Schier A. F. Malicki J.. (1996). Mutations affecting development of the notochord in zebrafish. Development 123, 117–128. 9007234 10.1242/dev.123.1.117

[B47] Strazielle N. Ghersi-Egea J. F. (2000). Choroid plexus in the central nervous system: biology and physiopathology. J. Neuropathol. Exp. Neurol. 59, 561–574. 10901227 10.1093/jnen/59.7.561

[B48] Strazielle N. Preston J. E. (2003). Transport across the choroid plexuses *in vivo* and *in vitro*. Methods Mol. Med. 89, 291–304. 10.1385/1-59259-419-0:291 12958428

[B49] Sykes D. Sweet D. H. Lowes S. Nigam S. K. Pritchard J. B. Miller D. S. (2004). Organic anion transport in choroid plexus from wild-type and organic anion transporter 3 (Slc22a8)-null mice. Am. J. Physiol. Renal Physiol. 286, F972–F978. 10.1152/ajprenal.00356.2003 15075193

[B50] Thambisetty M. Simmons A. Velayudhan L. Hye A. Campbell J. Zhang Y.. (2010). Association of plasma clusterin concentration with severity, pathology, and progression in Alzheimer disease. Arch. Gen. Psychiatry 67, 739–748. 10.1001/archgenpsychiatry.2010.78 20603455 PMC3111021

[B51] Tsuneki K. (1986). A survey of occurrence of about seventeen circumventricular organs in brains of various vertebrates with special reference to lower groups. J. Hirnforsch. 27, 441–470. 3760554

[B52] Umans R. A. Taylor M. R. (2012). Zebrafish as a model to study drug transporters at the blood-brain barrier. Clin. Pharmacol. Ther. 92, 567–570. 10.1038/clpt.2012.168 23047649 PMC5706651

[B52a] Urasaki A. Morvan G. Kawakami K. (2006). Functional dissection of the *Tol2* transposable element identified the minimal *cis*-sequence and a highly repetitive sequence in the subterminal region essential for transposition. Genetics 174, 639–649. 10.1534/genetics.106.060244 16959904 PMC1602067

[B53] Vercellino M. Votta B. Condello C. Piacentino C. Romagnolo A. Merola A.. (2008). Involvement of the choroid plexus in multiple sclerosis autoimmune inflammation: a neuropathological study. J. Neuroimmunol. 199, 133–141. 10.1016/j.jneuroim.2008.04.035 18539342

[B54] Villalobos A. R. Miller D. S. Renfro J. L. (2002). Transepithelial organic anion transport by shark choroid plexus. Am. J. Physiol. Regul. Integr. Comp. Physiol. 282, R1308–R1316. 10.1152/ajpregu.00677.2001 11959670

[B55] White R. M. Sessa A. Burke C. Bowman T. Leblanc J. Ceol C.. (2008). Transparent adult zebrafish as a tool for *in vivo* transplantation analysis. Cell Stem Cell 2, 183–189. 10.1016/j.stem.2007.11.002 18371439 PMC2292119

[B56] Wijnholds J. Delange E. C. Scheffer G. L. Van Den Berg D. J. Mol C. A. Van Der Valk M.. (2000). Multidrug resistance protein 1 protects the choroid plexus epithelium and contributes to the blood-cerebrospinal fluid barrier. J. Clin. Invest. 105, 279–285. 10.1172/JCI8267 10675353 PMC377447

[B57] Winata C. L. Korzh S. Kondrychyn I. Zheng W. Korzh V. Gong Z. (2009). Development of zebrafish swimbladder: the requirement of Hedgehog signaling in specification and organization of the three tissue layers. Dev. Biol. 331, 222–236. 10.1016/j.ydbio.2009.04.035 19422819

[B58] Wodarczyk C. Rowe I. Chiaravalli M. Pema M. Qian F. Boletta A. (2009). A novel mouse model reveals that polycystin-1 deficiency in ependyma and choroid plexus results in dysfunctional cilia and hydrocephalus. PLoS ONE 4:e7137. 10.1371/journal.pone.0007137 19774080 PMC2743994

[B59] Wolburg H. Paulus W. (2010). Choroid plexus: biology and pathology. Acta Neuropathol. 119, 75–88. 10.1007/s00401-009-0627-8 20033190

[B60] Xie J. Farage E. Sugimoto M. Anand-Apte B. (2010). A novel transgenic zebrafish model for blood-brain and blood-retinal barrier development. BMC Dev. Biol. 10:76. 10.1186/1471-213X-10-76 20653957 PMC2914679

[B61] Zhang J. Piontek J. Wolburg H. Piehl C. Liss M. Otten C.. (2010). Establishment of a neuroepithelial barrier by Claudin5a is essential for zebrafish brain ventricular lumen expansion. Proc. Natl. Acad. Sci. U.S.A. 107, 1425–1430. 10.1073/pnas.0911996107 20080584 PMC2824400

