# Supplementary material for: Functional and genetic analysis of choroid plexus development in zebrafish
Source: Front Neurosci. 2014 Nov 10;8:364. doi: 10.3389/fnins.2014.00364 (PMC4226144; doi:10.3389/fnins.2014.00364)
Supplement: Supplementary file 8 [file DataSheet1.DOCX]

***Supplementary Material***

**Functional and Genetic Analysis of Choroid Plexus Development in Zebrafish**

**Hannah E. Henson^1,2^, Chaithanyarani Parupalli^1^, and Michael R. Taylor^1^***

^1^Dr. Michael R. Taylor Laboratory, St. Jude Children’s Research Hospital, Chemical Biology and Therapeutics, Organization X, Memphis, TN, USA

^2^University of Tennessee Health Science Center, College of Graduate Health Sciences, Integrated Program in Biomedical Sciences, Memphis, TN, USA

*** Correspondence:** Michael R. Taylor, Michael R. Taylor Laboratory, St. Jude Children’s Research Hospital, Chemical Biology and Therapeutics, 262 Danny Thomas Place, Memphis, TN, 38105-3678, USA.

[Michael.Taylor@stjude.org](mailto:Michael.Taylor@stjude.org)

1. **Supplementary Movies**

**1.1 Supplemental Movie 1**

Confocal time lapse imaging of *Et(cp:EGFP)^sj2^* larvae to observe CP development. Time lapse was initiated at approximately 30 hpf and images were acquired every 30 min for 65 h. The image is a dorsal view at 20× magnification. CP epithelia are represented by GFP expression in green. File is available for download as a wmv.

**1.2 Supplemental Movie 2**

Confocal time lapse imaging of wild-type *Et(cp:EGFP)^sj2^* larvae. Time lapse was initiated at 54 hpf and images were acquired every 30 min for 48 h. The image is a dorsal view at 20× magnification. CP epithelia are represented by GFP expression in green. File is available for download as a wmv.

**1.3 Supplemental Movie 3**

Confocal time lapse imaging of *cp140.2* mutant larvae. Time lapse was initiated at 54 hpf and images were acquired every 30 min for 48 h. The image is a dorsal view at 20× magnification. CP epithelia are represented by GFP expression in green. File is available for download as a wmv.

**1.4 Supplemental Movie 4**

Confocal time lapse imaging of *cp9.6* mutant larvae. Time lapse was initiated at 54 hpf and images were acquired every 30 min for 48 h. The image is a dorsal view at 20× magnification. CP epithelia are represented by GFP expression in green. File is available for download as a wmv.

**1.5 Supplemental Movie 5**

Confocal time lapse imaging of *cp79.6* mutant larvae. Time lapse was initiated at 54 hpf and images were acquired every 30 min for 48 h. The image is a dorsal view at 20× magnification. CP epithelia are represented by GFP expression in green. File is available for download as a wmv.

**1.6 Supplemental Movie 6**

Confocal time lapse imaging of *cp151.2* mutant larvae. Time lapse was initiated at 54 hpf and images were acquired every 30 min for 48 h. The image is a dorsal view at 20× magnification. CP epithelia are represented by GFP expression in green. File is available for download as a wmv.

**1.7 Supplemental Movie 7**

Confocal time lapse imaging of *cp27.5* mutant larvae. Time lapse was initiated at 54 hpf and images were acquired every 30 min for 48 h. The image is a dorsal view at 20× magnification. CP epithelia are represented by GFP expression in green. File is available for download as a wmv.
